# Actual, Not Text-Book, Experience with Cases of Ectopic Gestation1Read by invitation before the Medical Society of the County of Erie, at Buffalo, June 14, 1892.

**Published:** 1892-08

**Authors:** James F. W. Ross

**Affiliations:** Toronto; Fellow of the American Association of Obstetricians and Gynecologists


### ACTUAL, NOT TEXT-BOOK, EXPERIENCE WITH CASES OF ECTOPIC GESTATION.[1]

BY JAMES F. W. ROSS, M. D., Toronto.

Fellow of the American Association of Obstetricians and Gynecologists.

IF YOU desire to know whether a woman hath conceived or not, give her, going to rest, a draft of metheglin, and if afterwards she feels pains in her belly caused by wind, she hath conceived; if none, she hath not.

Aphorisms of Hippocrates.

Again,—if a woman doth not conceive, and you are desirous to know whether she is capable or not, wrap her close around with clothes and put a perfume under her, and if she perceive the scent to pass through her body to her nose and mouth, be assured it is not her fault if she is barren.

Aphorisms of Hippocrates.

The great boon of a practitioner in these odorous days of surgery ought to be a wife who cannot smell, but, according to Hippocrates, one dare not select such a wife if he must choose between unfruitful union or an iodoform inconvenienced wife, unless he is prepared to shoulder the stigma of barrenness himself.

One must, however, admire such an acute observer as Hippocrates, though he gave us much that was inaccurate. I hope no text-book compiler will re-quote the above quotations, and hand them down as gospel from generation to generation. And yet this is done by compilers of text-books. I take it that all text-books should be compilations by many original workers in special fields, each writing only on the subject with which he is thoroughly familiar by virtue of a rich and fortunate experience. But the craze seems to be in direct opposition to this view. The man of least practical experience often writes the largest text-book. How are text-books written? A stenographer is engaged, old text-books are arranged around a room open at chapters on the same subject. These have all been previously reviewed and read by stenographer and author. The author now picks out a little from one, and a little from another, and endeavors to change as much as possible the wording so as to avoid any charge of piracy. Books are, therefore, filled with this "that may occur" and that "that may be given," and something else "that has been recommended," until the reader is either confused or forced to wade through a lot of padding that his own experience tells him is useless, before he is able to pick up some little crumb that may be of value.

---

1. Read by invitation before the Medical Society of the County of Erie, at Buffalo, June 14, 1892.

For many years the text-books did not recognize such a disease as pregnancy occurring outside of the uterus. When they did recognize it, the authors were unprepared to deal with it on surgical principles.

It has been said of a woman who floods to death, "extremam fundet sum sanguine vocem," and it is indeed so,— she casts forth with her blood her last breath; she dies from a misfortune, not a disease, from the result of a condition not quite normal.

The woman who dies from the hemorrhage resulting from an ectopic gestation is similarly situated. In the one case every effort is made to save life, to stop the torrent; in the other the surgeon sits passively by — yes, even today sits passively by — and watches the life ebb out. Barnes came boldly to the front and asserted that the day would come when a surgeon of strong convictions would cut down and tie the bleeding tube, just as any one worthy the name of surgeon would cut down and tie a bleeding femoral artery.

Lawson Tait began to do pioneer work, and his enemies must admit, no matter how distasteful it may be to them, that his teaching has already saved several hundred lives. In my own experience, five out of six lives have been saved by following Tait's lead. I have operated on seven cases of extrauterine pregnancy with only one death, and that of a woman in the last stage of septic poisoning, after five weeks of intraperitoneal suppuration. One case having reached full time, cannot be classed among the lives saved by the procedure so boldly carried out by Tait. I have assisted a colleague to operate on a case positively diagnosticated by us as one of unruptured extrauterine pregnancy. It was an unruptured extrauterine pregnancy. But I have also operated on two cases in which I excluded extrauterine pregnancy, and they both proved to be cases of ruptured ectopic gestation. I saw Mr. Tait operate on three cases. I have also seen two other cases operated on by one of my Toronto colleagues. My experience is, therefore, limited to:

Ross 7, 6 recovered; Tait 3, 2 recovered; Temple 3, 2 recovered. Total number of cases, 13; number of cases recovered, 10.

I also saw Price operate on one case, but do not know the result. You must not, therefore, expect too much from me. I might write a book on the subject on the lines laid down above, but intend to confine myself to points gained by actual experience.

I realize the fact that I am now talking to general practitioners, the prototypes of "the sun, around which all other luminaries, each moving in its own little sphere, revolves," as my friend Dr. Gihon has so well put it. If the general practitioner will wade through the literature on this subject, he may probably find that the symptoms of ectopic gestation can be exhaustively collated as follows :

*History of Labors.*—In most cases there has been a considerable period of sterility preceding the ectopic gestation.

*History of Tubal or Pelvic Disease.*—This will usually have been present. The history often shows that the patient has had a bad miscarriage some time before, or a supposed recent miscarriage.

*Menstrual History.*—May, perhaps, have missed a month, or a period has been anticipated, scanty, or delayed a day or two. Then irregular discharges of blood have begun, and sometimes been so severe and dangerous to the patient as to require plugging. Sometimes these hemorrhages will come on two, three, or four times in a month. In some they will be almost continuous. This discharge, with the passage of decidua, makes the patient assert positively that she has had a miscarriage.

*Pain.*—Some have no pain. Others have dysuria, or frequent desire to micturate, a desire to strain, a feeling as if something were coming down. Pains may be paroxysmal, like labor pains, or like the pains of a miscarriage, and they may be accompanied by a gush or a discharge of blood.

*Breasts.*—Some have shooting pains in the breasts, with a hot feeling. Breasts may tingle and feel full. Milk may be present.

*Nausea.*—Evidently not a reliable symptom.

*Other Feelings.*—May believe herself pregnant, or the reverse, but such feelings cannot be relied on.

*Examination.*—Vagina may be of a purplish color. The cervix, perhaps, is soft and patulous. Uterus may be pressed forwards or backwards, to one side or the other. An irregular swelling that feels like no other swelling, neither like a pus tube or ovary, that feels knotty and boggy, will be found in its neighborhood. Some say the tumor may pulsate. No placental souffle can be made out at this early period, even with the stethoscope in the vagina.

These will be the symptoms of this condition before rupture or suppuration. Though a general practitioner has no particular interest in anything but the diagnosis and treatment of this condition,

I would like to give the reasons for the foregoing symptoms so that they may be more readily brought forward on the spur of the moment at the sick bed.

The very cause of the sterility is the cause of the fertilization of the ovum and its non-removal from the tube. For many years the egg has managed to escape fertilization, but, at last, one of the spermatozoa, more vigorous perhaps than his fellows, has reached the egg and the damage is done. Many of these patients contract gonorrhea soon after marriage, the tubes are affected, but only to a minor degree ; the ciliated epithelium is shed, and the tube is functionally imperfect. The supposed miscarriage always requires rigid scrutiny. Close cross-examination will generally elicit the fact that the doctor said it was a false pregnancy, or that he thought it was a miscarriage, although unable to find a fetus. The discharge of blood is due to the separation of the decidua. This forms exactly as it does when a genuine pregnancy occurs. This bleeding from a uterus that is next door neighbor to a pus tube or ovarian cyst, is nothing unusual. It frequently occurs after abdominal operations and may even occur into the vagina from the stump of cervix left after hysterectomy. This symptom cannot, therefore, be relied upon. It is frequently entirely absent. As to pain, the dysuria, dyspareunia, dyschezia, are the accompaniments of so many other intrapelvic conditions that they cannot be relied on in this connection. Pain may indicate rupture of the tube and hemorrhage, or such hemorrhage may occur with scarcely noticeable pain. The presence of the apparent foreign body will of itself produce pain that is likely to attract the patient's attention, but it is not severe. This pain is of very great importance. The woman has been practically a well woman until it set in. What diseases produce such pain in a healthy woman ? There is no evidence or history of recent gonorrhea, though there may be of old infection. The woman has had no miscarriage ; she has had no intermittent attacks of pelvic inflammation, but while progressing well, though barren, she suddenly, in the midst of health, becomes an ill woman. Ectopic gestation is the only disease that will produce this condition. It is often put down to sweeping the house, doing a day's washing, straining at stool, and a hundred and one other causes physiologically and pathologically unexplained, but will be found by the abdominal surgeon to be due to the presence of an ectopic gestation. I am now speaking from actual experience. In whist, we say when in doubt "play trumps;"

well, when dealing with pelvic diseases, diagnose by exclusion, and when you meet a peculiar puzzling condition that feels neither like a pus tube nor cystic ovary, that is too soft for a fibroid with a pedicle, that is too hard and too movable for a hydrosalpinx, that does not feel in shape like an ovary, but is too hard for a tube, that takes some eccentric position between uterus and bladder and simulates that disease of the ancients, "pelvic cellulitis"—then make a guess,—guess ectopic gestation, and from actual experience I know that your guess will probably be correct.

Do not rely on breast symptoms, or sickness at the stomach, or peculiar longings, or a woman's own ideas of her condition, or you will find that you have trusted in vain. In no cases must greater care be observed in eliciting all the facts,—the real, not the imaginary facts. Leading questions must be avoided or the symptoms can be made to suit every pelvic disease. I have been astonished at the different aspects borne by a history read before and then after operation ; I have chided myself with my own blindness. When such puzzling cases occur, the history should be carefully written on a sheet of paper. At a subsequent visit this should be produced and a second cross-examination indulged in to see that the patient tells the same story over again. We are thus able to verify the original story.

After some authorities laid down definite symptoms as indicative of ectopic gestation, cases with similar symptoms were diagnosticated as cases of ectopic gestation, and were in more than one instance operated upon. Such cases were found occasionally to be cases of normal pregnancy. I cannot see why the two conditions should be confused if the physician carefully analyzes all the symptoms. After the profession felt assured that the diagnosis was easy and certain, that supposed cure-all, electricity, was introduced to kill the fetus and destroy the activity of the placenta. But many an ectopic pregnancy has disappeared without electricity, and, in fact, without any treatment, and certainly without any correct opinion of the case having been arrived at.

We have affecting women a disease called pelvic abscess. I am convinced that many such cases are not ones of pelvic abscess, in the ordinary acceptation of the term by the profession, but they are cases of old extrauterine pregnancy, with intraperitoneal hemorrhage and secondary suppuration in the clot. This suppuration may not tolerate the prescribed bounds of a jealous peritoneum, and we then have resulting general purulent peritonitis.

As to diagnosis. Can the rupture of an ectopic gestation give rise to symptoms that will point to the rupture? Very frequently they will not. A woman may be going around with blood in her abdomen without showing any definite signs of the presence of such blood; and yet peritonitis may supervene at any moment.

Such a peritonitis to me indicates the commencement of suppuration, and the indication is immediate operation. The temperature and pulse will be elevated in such a case. The temperature after rupture may remain normal for some time until the final explosion takes place and suppuration sets in. The woman is then generally in a good condition for operation, but if operation be delayed, her life will be sacrificed. Before such rise of temperature occurs, even though rupture has taken place, many cases will do well if allowed to wait. Absorption will occur, and when it does occur subsequent to the adverse opinion of a consultant, the one giving the adverse opinion feels that he has been wanting in judgment. He will feel repaid for this, however, when he thinks of the numerous occasions on which his judgment was not wanting, but when, after advising operation, his advice was rejected and the patient died.

Hematocele is nearly always connected with ruptured tubal pregnancy. After washing out the hematocele, indications of extra-uterine pregnancy will be apparent to the observer accustomed to see cases of ectopic pregnancy.

I show a specimen from a woman who had an enormous suppurating hematocele. The fetal sac was almost overlooked, lying, as it did, high up among the intestines.

Sudden symptoms simulating acute poisoning are at times met with. In my own cases, though the vomiting was severe, I never for an instant suspected poisoning.

In Case No. I. I diagnosticated the condition before operation, but this was two weeks after full term.

Case No. II. I diagnosticated a ruptured tube or ovary produced by my examination.

Case No. III. Had well marked symptoms of ectopic gestation, but I hesitated about admitting its presence.

Case No. IV. Nonplussed; could not diagnose the mass as anything definite; I think that given such a case again I would, by the process of exclusion, favor a diagnosis of ectopic gestation.

Case No. V. I diagnosticated this as suppurating hematocele, and leant toward ectopic gestation as the cause of the hematocele (as I always do).

Case No. VI. Nonplussed; diagnosticated, after rupture into the peritoneal cavity of a suppurating clot due to ectopic gestation as a ruptured pus tube.

Case No. VII. Not yet reported, but positively diagnosticated as secondary suppuration in the clot poured out by a hemorrhage due to ectopic gestation.

The case of this woman was so typical that I will briefly relate it.

Mrs. X., aged about 31 years. Her attendant physician told me that he attended the husband with gonorrhea about the time of his marriage; that the wife contracted the disease and suffered what he now believes was inflammation of the tubes. She was then sterile for three years, though otherwise in good health. A period was missed by three weeks. Flowing then began and lasted for three weeks. Abdominal pain of an uncomfortable but not severe nature came on and lasted for two weeks. At the end of this time she was straining at stool, when sudden pain seized her low down in right side of lower abdomen. She managed to crawl to bed, and arising a few hours after, the pain recurred. Three times she made the attempt to walk about, and three times the pain came on. Her temperature and pulse were normal. They began to rise about five days after the first attack. May 11, 1892, pulse was 130, temperature, 102 2-5.

On opening the abdomen its lower part was found filled with blood—grumous, coffee-ground colored, stinking blood. The tubal pregnancy was shelled out and tied off, and the patient has made an excellent recovery.

I will not burden you with the details of the other cases. This paper is only intended to be a hurried, tumbling together of a few disconnected facts, the outcome of my own observation of a small number of cases. You may take them for what they are worth.

If, after cases have resisted all other methods of diagnosis, as they will do very frequently, the abdomens are opened, the true state of affairs will be found, and the treatment can then be tacked on to the tail end of the diagnosis, and a cure will result.

In all cases of obscure miscarriage with a doubt as to the previous abortion, with a mass in the pelvis and unaccountable pains, a consultation should be called before the patient lapses into a critical condition. An operation should then be performed, partly exploratory and wholly curative.

The results of such operations done early can compare favorably with the most successful operations known to modern surgery.

DISCUSSION.

Dr. M. A. Crockett: I am very glad of an opportunity to exhibit these specimens to the Society, in connection with Dr. Ross's paper. The three specimens which I have here illustrate the two extremes of ectopic gestation. The remains of this fetus (specimen No. 1) were removed by Dr. Mann after having been in the abdomen for fifteen years. The case had gone to full term, and the child died. The woman suffered no particular discomfort, married a second time, and had one or two children. Last Fall she noticed that seeds, etc., passed out with the urine, and she began to show evidences of disturbance in the sac. On section of the abdomen, three openings between small intestines and sac were found, also an opening from the sac into the bladder. The holes in bladder and intestines were closed and the fetus and sac removed. It evidently was a double-horned uterus, the pregnancy occurring in one cornu. The patient made a good recovery.

This second fetus was carried by the woman for seven or eight years, which then, for some reason, began to cause pain, fever, and general disturbance. Dr. Mann opened the abdomen, but found the sac universally adherent, so removed the fetus, and packed. The placenta was, apparently, still vascular, and came away gradually piecemeal. The patient recovered. You will note that the specimen is a well-developed, full-term fetus.

These cases well illustrate the long quiescence yet ever-threatening danger of such conditions, and, to my mind, are important reasons for early operation.

This third specimen represents the other end of the series. The patient was sent to the hospital by Dr. Mann, with a diagnosis of ectopic gestation. The history was typical, and just such a one as Dr. Ross describes. An apparently normal pregnancy, with sudden and severe attacks of pain, with collapse at about three and a half months. Owing to Dr. Mann's illness, the case passed into my hands. I opened the abdomen and removed this sac containing the fetus. It was easily shelled out, one or two adhesions were tied, but there was nothing like a pedicle. The patient did well. This mass is largely made up of fibrin. Apparently, rupture had taken place into the broad ligament. During the last two years we have had at the hospital six or seven cases like this, the operation in all cases being followed by recovery.

Dr. W. S. Tremaine: Having been called on by the president, I wish to say that I have been very much interested in the paper. I have had an opportunity of seeing a number of cases where operative interference had saved the patient, and am among those who prefer laparatomy to electricity in ectopic pregnancy. It was my privilege to see several of the cases with Dr. Mann, from which the specimens exhibited by Dr. Crockett were taken, notably one case occurring in the wife of a prominent citizen, where correct diagnosis and prompt operation, undoubtedly, saved the patient's life. Cases of extrauterine pregnancy may with propriety be divided into *acute* and chronic; in the former tentative or timid policy would result in the patient's death.

To one remark in Dr. Ross's paper I would take exception, namely, when he spoke of "that disease of the *ancients*, pelvic cellulitis." While recent investigations have unquestionably shown that many cases of pelvic inflammation originate from diseased tubes, it is a fact within my personal observation that by no means all of them originate in this way. The "cellular" or connective tissue is the great nutritive framework of the body, and the *tissue* of inflammation; but pelvic inflammatory products do not always require laparatomy to remove them. The subject is a wide one, and I would like to discuss it at length, but we have with us today several brilliant gentlemen from other large medical centers, whose wide experience and successful results entitle them to be recognized as experts, and I feel sure the society is anxiously waiting to hear from them, so I will no longer trespass upon its patience.

Dr. JOSEPH PRICE, of Philadelphia: It gives me pleasure to discuss Dr. Ross's valuable paper upon this very important subject. I have no criticism to make, and shall only attempt to fortify, from my own practice and the deductions of my own experience, the points he has ably presented and emphasized. I wholly agree with him. Our knowledge of the pathology and our confidence in what we can surgically accomplish are precisely the same. We agree as to our possibilities and limitations. Such papers should be read and discussed before every county society in the land. It is, in the very highest sense, missionary work. Their reading and discussion teach and impress those lessons through which hundreds of human lives are saved, and that the general practitioner first sees these cases, not the pelvic surgeon.

Probably, no element enters into these cases more vital in importance than that of promptitude. This lies largely, if not altogether, with the general practitioner. The consensus of opinion in the discussion of this subject is in strange and marked contrast with what it was a few years ago. Then the discussions were conducted under the Marquis of Queensbury rules instead of under Cushing's Manual of Parliamentary Practice. A number of valuable specimens have been presented today. They beautifully illustrate this good work now going on in every section of our country. The evidence is cumulative of our certain and rapid advances. If the specimen shown by Dr. Crockett, of thirteen years' standing, had been operated on at once, the woman would have been saved much suffering and great menace to her life. There is always danger in any such quiescent conditions, of renewed inflammation, perhaps of a septic nature, during labor. In one of my own cases, I was making an operation on what was supposed to be a large uterine fibroid. On introducing a corkscrew to facilitate its removal, fluid escaped, and investigation showed that I had to deal with an ectopic gestation. If it had not been for this accident, the specimen might have been put in a museum as an illustration of fibroid tumor. I mention it to show on what delicate pivots diagnosis and successful operation sometimes turn.

The frozen specimens of Hart and Barbour deserve careful study, and illustrate valuable lessons. One of the great dangers in operations for ectopic pregnancy is bowel adhesions, and every one who operates must be prepared to suture one or more of these. The tumor is usually tubal, and in a large experience, over 100 cases, including my own and those of my assistants and pupils, I have never found the ectopic sac between the leaflets of the broad ligament ; moreover, I wish to further assert that I am unacquainted, from a personal experience, with the condition called pelvic hematocele. I have never seen, at the operating table, a pure and simple specimen of pelvic hematocele as described in the books. This condition is resultant from ruptured tubal pregnancy, in my opinion, but is not to be described as a distinct pathological entity. I wish further to state that, with few exceptions, all of my cases of extrauterine pregnancy have been plainly and distinctly tubal in character, and this leads me to the conviction that it is practically the only form that this disease assumes. The exceptions are so few, that they have no practical interest at the operating table.

In 1790, Dr. William Bayne, of Virginia, first recognized the condition of extrauterine pregnancy, and verified his observations by successful operation. He repeated this in 1799, in a second case occurring in a slave on whom he also operated with good results. These two recoveries, made after diagnosis and skilful operation, have not been exceeded by any modern abdominal surgeon in their conception and execution. The work done by this distinguished Virginian laid the foundation for all future operative success, and entitles him to stand side by side in our memories with McDowell, who, in 1809, demonstrated to the world the propriety of operation in all cases of ovarian tumor.

In my opinion, there is no pelvic disease demanding abdominal section that offers such good prospects of success as ectopic pregnancy. I remember well the words of the late Dr. Agnew, with reference to hemorrhage in general, namely, "seek the offending vessel and tie it," and this as much as any other one factor induced me to operate in my first case. The urgency of operation in ruptured tubal pregnancy, to institute a comparison, is the same as in ruptured aneurism or a stab wound of the abdomen. In a case of doubt the exploratory incision need not even penetrate the peritoneum. If the peritoneum is black, there is intraperitoneal hemorrhage ; if it is normal in color, there is no need of incising it. A doughy, foreign mass, inaptitude to conception, missing of one or two menstrual periods or a delayed period, characteristic pain like that of old-fashioned "dry gripes," but more peculiarly intense and cramp-like, together with shock, are diagnostic points which may be relied upon as indicating ectopic gestation with rupture. We must exclude fecal accumulation when feeling the doughy mass alluded to. The shock in ruptured tubal pregnancy is greater than can be accounted for by hemorrhage alone. There is something almost mysterious about it, and I find it no where else so depressing. Most operators, in my experience, work too much at the anterior surface of new growths in the pelvis. I would advise and accentuate the importance of seeking in this operation a plane of cleavage high up and towards the sacrum.

One of my cases illustrates well the need of conservatism. I removed an ectopic sac from the cornu of the uterus and was about to plunge a corkscrew into the latter, but, finally, decided to leave it in place. In four or five months the patient gave birth to a fine healthy boy. This illustrates well both ectopic and normal pregnancy coëxisting in the same patient.

I have often referred to the late Dr. Formad's statistics when he was coroner's physician for Philadelphia. He found a large number of cases of hemorrhage in the pelvis among sudden deaths that he regarded as accidental, until gynecological surgeons taught him that they were due to ruptured tubal pregnancy. Of 35 such cases, 31 had died within a few hours after the appearance of unfavorable symptoms. Later Dr. Formad reported that operations for ectopic gestation had greatly lessened his post-mortem experience in that class of cases. Unruptured tubal pregnancy is rarely met with, but I wish to assert my belief in the propriety of operating in all cases where a diagnosis is made either before or after rupture. In all my operations for this malady, 76 in number, I have lost but two patients, and they were among my first eight cases ; I attribute the loss of these to bad surgery.

[Note by the Editor.—This is a very imperfect synopsis of Dr. Price's discussion, which was given at considerable length, and was accepted by the audience as a most instructive elaboration of the subject.]

Dr. F. W. Bartlett : Not intending to intimate that Dr. Price, in speaking of neglect to operate in this case (case No. 1 of Dr. Crockett's specimens), reflects upon the physicians who, from time to time, were consulted, I yet desire to briefly give the history of the same. The patient was seen by me in the early morning, after some hours' suffering from pains of parturition, a physician being in attendance. These had subsided. Examination proved to me that the gestation was ectopic, which opinion was confirmed by Dr. C. C. Wyckoff in consultation. Later the patient was referred to the care of the late Dr. James P. White, who advised operative interference, which was declined. The woman subsequently, being divorced from her husband, married again, and bore two children, but of this I knew nothing until called to see her on account of increasing discomfort in the abdomen. I at once requested a consultation with Dr. M. D. Mann, who recognized the condition and advised removal, but it was declined, and only yielded to when the symptoms described by Dr. Crockett were manifested. This statement seems just to all concerned.

Dr. Ross, closing the discussion: The very remarkable part of the behavior of one of the cases related by Dr. Crockett, is that the placenta remained active for such a long time after the death of the fetus. Surely one such case from an actual intra-abdominal observation, is worth more than hundreds of the mythical cases reported as ones of ectopic gestation and subsequent electrocution of the fetus. I defy any man to assert that such and such a case is one of ectopic gestation, and feel that it is impossible that he should be mistaken. I can diagnosticate a case of extrauterine pregnancy I think as well as the average of experts; I have done so, and have verified my diagnosis, but on a subsequent abdominal exploration I have been mistaken more than once. It is so easy to say, "oh! ectopic gestation," and to shoot a few electrical shocks through the supposed fetus. No knife is used, the patient is pleased, her life that was supposed, probably through an error, to be so much endangered, is apparently saved, and the man who is opposed to such operations is lauded to the skies as a wonder; the case is quoted and requoted, and used as a precedent in many a really critical case; the knife is then set aside and the next time a life is lost that might otherwise have been saved. These lives are not always lost at once or suddenly; they frequently rally from one, two, three or more attacks of hemorrhage, and then die of pelvic abscess at some later period. The difficulty is that even after rupture of the tube and hemorrhage, complete absorption may take place. But with such a safe procedure as operation at our command, it is very questionable whether we are justified in allowing our patients to avoid the risk of the knife, and thereby encounter risks, that, to my mind, are much greater.

No more eloquent appeal for early operation could be offered than this specimen, shown by Dr. Crockett. No criticism is meant of the treatment of Dr. Mann or Dr. Bartlett, because Dr. Mann did not see the case until a late date. The fault lay either with the patient or her first attending physician. Here were pieces of bone and flesh extruded, bit by bit, after a long illness. Now, take all the specimens, and speaking in a general surgical way (leaving all reference to the operators in the present cases out of the question), and with them compare the specimens I show removed at a very early date, and ask any unbiased thinker which shows the better surgery, the early or the late operation, and I think he will say, the early operation most decidedly. And yet, though any case left unoperated upon may become as formidable as any of these three cases operated on at a late date, we hesitate about what is supposed by many to be a terrible procedure, but is in reality a very safe surgical operation. As to the bleeding, I believe that it occurs from a rupture at the weak fimbriated end, in most cases, and not from rupture of the tube. Dr. Price has been led, so he informs me, after a very large experience, to come to the same conclusion.

My mention of pelvic cellulitis as a disease of the "ancients" has brought out criticism. I do not say that there is no such disease as pelvic cellulitis. It must, however, be a very curious and evasive disease, because it keeps out of the way of those who are constantly working inside of the abdomen. We never see it, but that fact, of course, does not prove that it never exists, and we are still waiting with alert eyes and fingers eagerly seeking conviction. It is strange how tenaciously men cling to the faith of their fathers. This faith in the "old" has its value, because a little obstruction to progress makes progress slower but surer and better. I admire many of these men, and am pleased to let them hold to their opinions, but I want to be allowed to hold to mine, and so do all of us who are working in this special field. We take nothing for granted, and accept as real only such facts as can be demonstrated to us, or that we can demonstrate to ourselves. Gynecology has merged into abdominal surgery, and the gynecologist who is not an abdominal surgeon, is still floundering in the dark, as the rest of us were a few years ago.

We have, unfortunately, many who are both inaccurate observers and timid operators, and these men, frequently speaking with what is considered the weight of authority, have a certain following. Their procedures are too long delayed and not sufficiently thorough, and the results are correspondingly disastrous.

